# Lifestyle Factors and Sleep Health across the Lifespan

**DOI:** 10.3390/ijerph18126626

**Published:** 2021-06-20

**Authors:** Joseph M. Dzierzewski, Sahar M. Sabet, Sarah M. Ghose, Elliottnell Perez, Pablo Soto, Scott G. Ravyts, Natalie D. Dautovich

**Affiliations:** Department of Psychology, Virginia Commonwealth University, Richmond, VA 23284, USA; sabetsm@mymail.vcu.edu (S.M.S.); ghosesm@mymail.vcu.edu (S.M.G.); pereze2@mymail.vcu.edu (E.P.); sotop2@mymail.vcu.edu (P.S.); ravytss@mymail.vcu.edu (S.G.R.); ndautovich@vcu.edu (N.D.D.)

**Keywords:** sleep health, lifestyle factors, aging, physical activity, screen time

## Abstract

Sleep health, operationalized as a multidimensional construct consisting of sleep regularity, satisfaction, alertness, timing, efficiency, and duration, is an emerging concept in the field of sleep medicine which warrants further investigation. The purpose of the present study was to: (1) compare sleep health across the lifespan, (2) determine lifestyle factors associated with sleep health, and (3) examine whether lifestyle factors associated with sleep health varied between and within age groups. Participants consisted of 3284 individuals (Mean age = 42.70; 45% male) who participated in a cross-sectional online survey of sleep and health. Sleep health was measured using the RU-SATED scale, while demographic and lifestyle factors (e.g., daily social media use, sedentary activity, fast food consumption, etc.) were all self-reported. Sleep health was the highest among older adults (*M* = 8.09) followed by middle-aged (*M* = 7.65) and younger adults (*M* = 7.16). Across age groups, fast-food consumption, daily regularity, and daily TV, social media, or internet use were all negatively correlated with sleep health (*p*s < 0.05). Few differences in the association between lifestyle factors and sleep health across age groups were found. Overall, these findings may help to inform sleep health promotion efforts by targeting the most pertinent lifestyle factors for promoting sleep health.

## 1. Introduction

Sleep is a critical health behavior, important for all aspects of well-being (e.g., see [1]). Although decades of research have focused on disordered sleep, including identifying important lifestyle factors [2,3,4] and age differences in poor sleep [5,6,7], comparatively less research has focused on healthy sleep. In fact, the concept of sleep health is a relatively new term in the sleep literature [8]. Generalizing findings from studies focused on poor and disordered sleep to sleep health may result in falsehoods and inaccuracies The present study examined sleep health across the age continuum, while investigating lifestyle factors that may be related to sleep health across the lifespan.

Sleep quality and quantity naturally change across the lifespan in complex ways, as evidenced by both self-report and objective assessments. Aging is often accompanied by changes in sleep routines and a decrease in sleep quality and quantity [7,9]. Although the need for sleep is stable throughout life, the ability to achieve adequate sleep decreases for many adults as they age [6]. Importantly, this decreased ability may not be the result of aging, but rather may result from other factors that accompany aging, such as medical conditions, poor physical health, increased medication use, and circadian rhythm disturbances [7,10,11]. However, findings are mixed in regards to the association between aging and sleep disturbances and related problems [12,13]. For example, numerous epidemiologic studies have revealed that sleep-related complaints and daytime sleepiness are more common among older adults in comparison with their younger counterparts [7,14]. By contrast, other studies have found that sleep-related complaints generally decline across the lifespan, with the fewest complaints endorsed by the oldest age groups (80+) after adjusting for sociodemographic factors, such as race, ethnicity, income, and education [15].

Nonetheless, sleep complaints in older adults are highly prevalent, including difficulty initiating or maintaining sleep, poor sleep efficiency, and daytime drowsiness [6,16,17]. Older adults experience an advanced circadian rhythm, whereby they feel sleepy earlier in the evening and awake earlier in the morning, and their ability to tolerate abrupt phase shifts worsens (e.g., from jetlag or nightshifts; [18,19]. In addition, sleep latency has been found to increase with age, while total sleep time, sleep efficiency, slow wave sleep, and rapid eye movement (REM) sleep have been found to decrease [6,7]. Although sleep-related disorders are common in the general adult population, the prevalence of these disorders increases with age [5,15]. Insomnia is the most common sleep disorder in adults and has a high overall prevalence in older adults [20,21]. Epidemiologic research indicates that older adults are at an increased risk of developing insomnia symptoms, which in turn is linked to a number of adverse health outcomes such as depression and poor cognitive functioning [16,22,23].

Similar to other medical disciplines, the area of sleep research has, for much of its history, focused on a narrow range of sleep problems (e.g., short sleep duration, difficulty falling asleep), disorders, and treatments. Although it is important to identify and treat sleep problems/disorders, sleep health is not simply the absence of a disorder [8]. The emerging concept of “sleep health” presents a more holistic view of sleep through a positive, health-oriented framework. According to Buysse (2014), sleep health includes the following critical dimensions of sleep: regularity, satisfaction, alertness, timing, efficiency, and duration [8]. Examining sleep health, and not exclusively focusing on disordered sleep, affords the opportunity to study sleep along a broader continuum that is inclusive of all individuals.

As sleep health has more recently entered the field, a relatively smaller body of work has focused on this construct. Additionally, because no universal definition of sleep health exists, research has assessed sleep health in a preliminary manner. These methods have focused on *individual* dimensions of sleep health or measurement of disordered sleep and inferred sleep health through the absence of sleep disorder symptomology. For example, studies have attempted to capture sleep health via a mixture of self-report and actigraphy assessed-sleep that reflect limited components of sleep health [24,25]. Studies employing dedicated sleep health measures have reported that greater sleep health is linked to social rhythm regularity and reduced mental health symptoms across the lifespan [26], and older age is associated with better sleep health [27].

Promoting sleep health has important clinical implications for sleep medicine and, more broadly, health and well-being. Multiple factors shape one’s sleep, including genetic, social, environmental, and behavioral domains. However, this research has predominantly focused on the individual components of sleep health (e.g., sleep duration, sleep efficiency) or disordered sleep. Given that many external lifestyle factors are largely modifiable (e.g., social media use, physical activity levels), understanding how these factors may be associated with sleep health could prove informative as they could be targeted and altered to promote sleep health. Although limited, there is evidence supporting an association between lifestyle factors and sleep health. A recent study found that being partnered and living in a higher income country were associated with better sleep health, while difficulties transitioning to working from home and maintaining a stricter level of quarantine during the COVID-19 pandemic were associated with poorer sleep health [27].

Much more work has examined lifestyle factors and sleep disturbance and/or specific sleep characteristics. For example, pet ownership has been linked to increased sleep duration and shorter sleep latency [28,29], whereas poor nutrition and food insecurity have been tied to shorter sleep duration, longer sleep latency, and a higher frequency in sleep complaints [30,31]. Similarly, increased exposure to media (e.g., TV, social media) has been linked to poorer sleep, whereby both short (<5.5 h) and long sleepers (≥8.5 h) have been found to watch more TV than the average sleeper [9]. In adolescence, higher usage of nighttime social media use has been linked to poorer sleep quality [4]. In adults across the lifespan, greater social media use was also associated with poorer sleep quality and shorter sleep duration, with the strength of this association increasing with age [32]. Additionally, reading a story via a printed book resulted in increased sleepiness at bedtime compared to reading the same story on a tablet, highlighting the consequences of blue light technology on alertness, sleep, and circadian rhythms [33]. Relatedly, irregularity of social rhythms has been linked to poor sleep in general [34], as well as sleep health specifically [26]. Exercise and sleep are both important health behaviors in adults across the lifespan and evidence for a reciprocal relationship exists in older adults [35]. Evidence suggests that increased activity levels in older adults improves sleep quality [36] and is protective against both incident and chronic insomnia [37]. Further, middle-aged adults who engaged in higher levels of moderate to vigorous physical activity were less likely to report a sleep disorder diagnosis compared with those who were less active [2]. Although many of these variables have been tied to sleep, the majority have yet to be studied in relation to sleep health.

The present study sought to fill gaps in the literature where there is a dearth of information focused exclusively on sleep health as an independent concept from disordered sleep. The first aim of the study was to examine age differences in reported levels of sleep health. Specifically, we sought to determine whether young adults, middle-aged adults, and older adults differed in their levels of sleep health. Next, we sought to determine how various lifestyle factors (e.g., dietary habits, owning pets, engagement in physical activity, time spent watching TV, time on social media platforms, time reading, time on the internet more broadly, overall proportion of the day being sedentary, and daily social rhythms), were associated with sleep health. Lastly, we investigated whether the associations between the aforementioned lifestyle factors and sleep health differed by age group. We hypothesized that older age would be associated with better sleep health, lifestyle factors typically associated with poor sleep would be inversely related to sleep health, and lifestyle factors would be similarly related across age groups and not differentially related within age groups.

## 2. Methods

### 2.1. Procedures and Participants

This study represents a secondary data analysis of participants who were enrolled in an online study examining sleep longitudinally across normal development (the ISLAND study). Individuals were required to be 18 years old and above, with access to a computer, tablet, or phone to participate in the study. Participants were subsequently categorized as young adults (aged 18–34 years), middle-aged (35–54 years), and older adults (55+ years).

Enrollment and participation in the study occurred via Amazon Mechanical Turk and Qualtrics. Participants were presented with information about the purpose of the study. All participants provided informed consent prior to their participation. A series of behavioral and psychological self-report questionnaires were completed by participants in exchange for USD 0.25. In order to maintain data validity, age consistency, and attention, checks were embedded within the series of questionnaires. The local Institutional Review Board approved all study methodologies.

### 2.2. Measures

Sleep health. Participants were asked to respond to the RU-SATED scale [8] as a measure of sleep health. Participants responded to 6 items related to sleep regularity, satisfaction, alertness, timing, efficiency, and duration broadly. Responses ranged from “Rarely/Never” to “Usually/Always” to such items as, “Are you satisfied with your sleep?” Higher summative scores on this scale are indicative of better sleep health. The scale has appropriate psychometric properties and is positively associated with self-rated sleep and sleep self-efficacy [38]. Internal consistency of the RU-SATED in the current sample was 0.64.

Fast food. Participants were asked to select the response that best represents their frequency of fast-food consumption. Responses to this item ranged from “I do not eat fast food” to “Nearly every day.”

Number of pets. Participants were asked to select the number that represents how many pets they own. The responses available in the pulldown menu ranged from “1” to “10+” pets.

Weekly moderate-to-vigorous physical activity. Participants were asked to provide an estimate of the number of days per week they engaged in moderate-to-vigorous physical activity (MVPA) using a pulldown menu. In addition, they were asked to provide an estimate for the amount of times per day dedicated to this level of physical activity. These two items were combined to provide an estimate of the amount of time (in minutes) of MVPA performed each week.

Daily percentage sedentary. Participants were asked to provide an estimate of the average percentage of their day spent sitting, including time during work and leisure. A visual analog scale was used to indicate the average percent of their day spent sedentary from “0%” to “100%.”

Daily social media minutes. Participants were asked to estimate the amount of time they spent daily on social media platforms, such as Facebook, Twitter, and Instagram. A numerical value of hours and minutes was provided by participants and converted to total daily minutes.

Daily internet minutes. Participants were asked to estimate the amount of time spent on the internet daily. Hours and minutes provided by participants were converted to total daily minutes.

Daily TV minutes. Similar to social media use and internet use, participants estimated the amount of time spent per day watching shows, movies, or television. All estimates were converted to total daily minutes.

Daily reading minutes. Participants provided an estimate for the amount of time they spent reading daily. All hours and minutes provided by participants were converted to total daily minutes.

Daily regularity. Six items from the 10-item Brief Social Rhythm Scale (BSRS) were used to measure regularity of engagement in basic daily activities. Basic daily activities assessed included regularity of mealtimes and time spent with others during free time. Four items pertaining to sleep were removed from the scale to avoid potential inflation of the association with sleep health outcomes. Responses to each item ranged from 1, indicating “Very Regularly,” to 6, indicating “Very Irregularly.” Higher total scores suggest greater irregularity of engagement in daily activities. Internal consistency of the BRBS in the current sample and a US sample were 0.81 and 0.83, respectively [39].

### 2.3. Analyses

A one-way ANOVA was utilized to examine potential age differences in sleep health across three age groups (young adult, middle-aged, and older adult). Bivariate correlations were used to evaluate the association between 9 different lifestyle factors and sleep health. In order to compare the strength of the correlation coefficients for the 9 lifestyle factors and sleep health, both between and within age groups, the obtained Pearson correlation coefficients underwent a Fisher’s Z transformation. Utilizing Fisher Z-transformed variables created a more normally distributed sample to accommodate comparison of the lifestyle factors across and within different age groups [40].

## 3. Results

### 3.1. Sample

Table 1 presents a detailed summary of demographic and lifestyle factors by total sample and by age group. Most participants in the current sample were White (80.8%) and were mostly evenly split between sexes (48.5% female, 45% male) and age groups (38.8% young adults, 31.8% middle age adults, 29.4% older adults). The majority of the sample did not own pets (36.6%). On average, participants spent about 5 h daily on the internet, 2.5 h daily watching television, 1.5 h daily on social media, and slightly over 1 h daily reading. Participants engaged in moderate-to-vigorous physical activity for slightly over 3 h per week on average, and just over half their day engaging in sedentary behaviors (62.5%). On average, participants endorsed moderate irregularity of engagement of basic activities. Frequency of fast-food consumption was typically once per week (36.1%) or once per month (35.2%).

### 3.2. Sleep Health Differences across the Lifespan

A one-way ANOVA was conducted to examine whether young adults, middle-aged adults and older adults differed in reported sleep health. The data violated the assumption of homogeneity of variances, as assessed by the Levene’s statistic (*p* = 0.02). There was a statistically significant difference in sleep health scores between the three groups, Welch’s *F*(2, 2115.748) = 33.425, *p* < 0.001. Sleep health in older adults (*M* = 8.09, *SD* = 2.62) was significantly better than sleep health in middle-aged (*M* = 7.65, *SD* = 2.76) and young adults (*M* = 7.16, *SD* = 2.62). Games-Howell post hoc analyses showed that mean differences between young adults and middle-aged adults (0.49, 95% CI [−0.75, −0.22]), young adults and older adults (0.92, 95% CI [0.66, 1.18]), and middle-aged adults and older adults (0.44, 95% CI [0.15, 0.72]) were all statistically significant.

### 3.3. Lifestyle Factors and Sleep Health across Age Groups

Pearson correlation coefficients were calculated to assess the associations among the nine lifestyle factors and sleep health across three separate age groups. Significant negative correlations were observed in young adulthood between sleep health and the following: weekly fast food intake (*r* = −0.135), total number of pets (*r* = −0.063), minutes of TV use (*r* = −0.132), minutes reading (*r* = −0.066), minutes of social media use (*r* = −0.131), minutes of general internet use (*r* = −0.152), and overall daily regularity (*r*= −0.320). Significant negative correlation coefficients between sleep health and lifestyle factors in middle adulthood emerged for the following: weekly fast food intake (*r* = −0.126), minutes of TV use (*r* = −0.171), minutes reading (*r* = −0.111), minutes of social media use (*r* = −0.196), minutes of general internet use (*r* = −0.233), and overall daily regularity (*r* = −0.340), along with a significant positive correlation between sleep health and weekly MVPA (*r* = 0.075). Lastly, in older adults, there were significant negative correlations between sleep health and fast food intake (*r* = −0.135), daily sedentary activity (*r* = −0.102), daily minutes of TV use (*r* = −0.129), minutes of social media use (*r* = −0.163), minutes of general internet use (*r* = −0.093), and overall daily regularity (*r* = −0.283), along with a significant positive correlation between sleep health and weekly MVPA (*r* = 0.090). See Table 2 for a full listing of correlations among sleep health and lifestyle factors across age groups.

### 3.4. Comparisons of Life Style Factors—Sleep Health Associations

Pearson correlations were transformed to Z-scores, denoted as Zr, utilizing published protocols [40]. This allowed for the comparison of lifestyle factors and sleep health both across and within different age groups. Comparisons between young adults and middle-aged adults revealed a significant difference in the strength of the association between sleep health and number of minutes spent on the internet (Zr = 2.014), with middle-aged adults displaying a stronger negative association than their younger counterparts. When comparing lifestyle factors between young adults and older adults, the groups only differed with respect to the strength of the association between sleep health and daily percent sedentary activity (Zr = −1.904). Sedentary behavior was more strongly related to sleep health in late-life than in young adulthood. Comparisons between middle-aged and older adults revealed two significant differences in the strengths of the associations between sleep health and daily minutes spent reading (Zr = −1.978) and daily minutes on the internet (Zr = −3.223). Middle-aged adults had stronger associations between these lifestyle factors and sleep health than older adults. See Table 3 for a full listing of between-age comparisons of the associations among sleep health and lifestyle factors.

The strength of associations between sleep health and MVPA minutes and sleep health and daily sedentary percent differed for both middle-aged (Zr = 2.611) and older adults (Zr = 3.691), but not young adults. However, the strength of associations between sleep health and daily social media use and sleep health and daily internet use only differed in older adults (Zr = −1.829), but not younger or middle-aged adults. See Table 4 for a complete listing of within group differences between sleep health and various lifestyle factors.

## 4. Discussion

The purpose of the current study was to examine age differences in sleep health and the roles of different lifestyle factors in relation to sleep health across, and within, young, middle-aged, and older adulthood. First, although prior research on components or disorders of sleep has suggested that sleep may worsen with age, older adults in the current study reported the best sleep health. Specifically, all three age groups significantly differed in their reports of sleep health, with sleep health higher in middle adulthood compared to young adulthood and in older adulthood compared to middle adulthood. This finding highlights the importance of considering sleep across a continuum (e.g., examining sleep health) versus solely focusing on disordered or impaired sleep, and is consistent with a small body of prior research suggesting that sleep may improve across the lifespan, especially when considering mediating factors like physical and mental health [15]. We found that behaviors traditionally evidenced to have an association with poor sleep, such as poor diet [30], sedentary behavior [2], daytime activity irregularity [34], and daily exposure to media [4,9], were associated with poorer sleep health across the lifespan within the present sample. Lastly, lifestyle factors, such as weekly fast-food intake, daily TV minutes, social media usage, internet usage, and daily regularity were negatively associated with sleep health across age groups. However, there were notable differences in the association of lifestyle factors with sleep health across age groups, including the number of pets, MVPA minutes, daily time sedentary, reading minutes, and internet minutes.

The number of pets owned was inversely related to sleep health in young adults but not middle-aged or older adults. Pet ownership’s association with sleep health findings may be dependent on the number of pets, type of pets, and level of pet attachment [28]. Younger adults may be new to pet ownership, while middle-aged and older adults may have had longer experience adjusting to and being attached to pets, thus reducing any potential associations. Contrary to our findings, pet ownership has been shown to be associated with positive health outcomes, including total sleep time, in older adults [29]. Importantly, the present study did not assess the impact of pets on sleep routines, such as bed-sharing, disrupting sleep, or promoting sleep via extra outdoor exercise.

Higher weekly MVPA minutes were associated with better sleep health in middle-aged and older, but not younger, adults. These findings are consistent with past literature suggesting that exercise has a meaningful impact on sleep in middle-aged and older adults compared to younger adults [41]. Additionally, daily percentage of time sedentary was inversely associated with sleep health in older adults, but not younger or middle-aged adults, with older adults demonstrating a statistically stronger association than younger adults. Notably, older age and retirement status are known determinants of amount of time spent sedentary [42].

Daily reading minutes were significantly inversely associated with sleep health in younger and middle-aged, but not older, adults and this association was statistically stronger in middle-aged adults as compared to older adults. Older adults may be more likely to read paper books rather than electronic books on e-readers which may emit blue light that can alter sleep characteristics. Indeed, reading from an e-reader or IPad has been shown to contribute to decreased subjective and objective EEG-measured sleepiness in comparison to reading from a printed book. Reading from a printed book has further been shown to elicit increased sleepiness, which may explain why reading minutes was associated with sleep health in younger and middle-aged, but not older adults in the present study [33].

The present study provides novel contributions to the research on lifestyle factors and sleep across the lifespan. Specifically, this study highlights differential associations between various lifestyle factors and sleep health between and within age groups. Alongside adding to a limited body of research specifically examining sleep health, the current study supports previous studies pointing to biopsychosocial factors, rather than solely age, contributing to poor sleep [7,10]. Further, through utilization of an outcome measure of sleep health, rather than sleep disturbance, the present study begins to fill in the gaps left by past research on lifestyle factors and sleep disturbance. A focus on sleep health shifts attention from correlates of poor sleep to modifiable targets in the preservation of sleep wellness. Indeed, by utilizing a measure of sleep health, rather than sleep dysfunction, we hope to broaden the current understanding of the association between lifestyle factors and sleep.

Beyond theoretical contributions to the literature, there are notable clinical applications following from this research that are worth consideration. The present study draws attention to the importance of an interdisciplinary approach to sleep medicine and sleep hygiene (e.g., nutrition, exercise, daily regularity). Further, the current study draws attention to the importance of assessing sleep health correlates by age, rather than taking a one-size-fits-all approach to assessment and treatment recommendations. When assessing sleep health, health care providers should assess for certain lifestyle factors either across or within age groups, with specific attention to those lifestyle factors that are more relevant for each age group individually. For instance, results suggest that fast-food intake, internet use, regularity, and social media use are meaningful contributors to sleep health across age groups. As such, these lifestyle factors can be part of a standard battery of questions assessing sleep health. As pet ownership, technology use, and reading minutes evidenced significant associations with sleep health in younger adults, these factors should be of high importance in assessment alongside a more general battery for younger adult patients of health providers. Similarly, MVPA minutes, percent sedentary, technology use, and reading minutes are noteworthy and foci worthy of consideration in the evaluation of middle-aged adult sleep health. Lastly, health providers should assess physical activity and levels of sedentary behavior in older adults when evaluating sleep health [36,41,42].

There are several limitations in the current study which must be addressed. The cross-sectional design limits the ability to assess causality or directionality of the associations of interest with sleep health. Longitudinal evidence is needed to examine the direction of associations between these factors and sleep health. Next, our measures of sleep health and lifestyle factors were all self-report in nature and are therefore subject to common issues associated with recall bias. Incorporating objective measures of physical activity and sleep (e.g., daily diary; actigraphy), would complement and enrich the measurement of these health behaviors. Measurement of daily social media and internet use via diaries or electronic means may also provide a more accurate and comprehensive view of usage than single-item retrospective recall estimates. Similarly, several questions focused on frequency to the omission of quantity, or vice-versa. A complete approach to measurement would assess for both frequency and quantity in all relevant domains. The current sample was rather homogenous with regards to race/ethnicity. Future investigations should take place in more diverse samples (e.g., with respect to race/ethnicity, gender, socio-economic status, disability status, etc.), to add to our understanding of how external factors are tied to sleep health across diverse groups. Finally, when considering lifestyle factors, it would be helpful to consider their relative contributions to daily activity. For example, the addition of any activity means there is less time for other activities. As such, reading, for example, may be detrimental for sleep because it replaces an activity that is beneficial like outdoor exercise. Continued examination of lifestyle factors and sleep health, through a socio-ecological framework (e.g., see [1]), will allow for the expansion of our understanding of these complex associations.

Overall, the present study provides initial evidence for the importance of a variety of lifestyle factors for sleep health outcomes across the lifespan. Findings suggest that although there are certain factors that are associated with sleep health across age groups (e.g., exercise, diet, regularity), there are important inter- and intragroup differences that warrant further consideration and exploration. Future research should attend to underlying mechanisms linking lifestyle factors and sleep health in order to broaden our understanding of these important associations and inform potential lifestyle interventions for sleep health across the lifespan.

## Figures and Tables

**Table 1:** Participant Descriptive Statistics.

Variables, Mean (SD)				
	Full Sample	Young Adults	Middle Age Adults	Older Adults
	(N = 3284)	(N = 1274)	(N = 1045)	(N = 965)
Age	42.7 (16.7)	25.8 (4.3)	43.6 (6.0)	64.2 (6.7)
Gender, N (%)				
Female	1594 (48.5)	569 (44.7)	511 (48.9)	514 (53.3)
Male	1479 (45.0)	533 (41.8)	503 (48.1)	443 (45.9)
Non-binary	66 (6.4)	172 (13.5)	31 (3.0)	8 (0.8)
Race, N (%)				
White	2652 (80.8)	935 (73.4)	875 (83.7)	842 (87.3)
Black	263 (8.0)	120 (9.4)	76 (7.3)	67 (6.9)
Latinx	216 (6.6)	123 (9.7)	60 (5.7)	33 (3.4)
Asian	208 (6.3)	126 (9.9)	53 (5.1)	29 (3.0)
Other race	122 (3.8)	62 (4.9)	35 (3.3)	25 (2.5)
Daily Activities				
Daily TV ^a^	153.1 (122.1)	145.3 (120.8)	141.1 (109.7)	176.2 (133.2)
Daily social media ^a^	91.3 (95.9)	119.5 (113.5)	79.2 (80.7)	67.1 (73.7)
Daily internet ^a^	311.0 (172.3)	343.5 (176.2)	302.7 (174.0)	277.0 (157.3)
Daily reading ^a^	74.8 (79.0)	71.7 (84.9)	67.7 (72.6)	86.8 (76.1)
Weekly MVPA ^a^	196.4(254.9)	209.5 (264.6)	185.3 (242.8)	191.1 (254.3)
Daily % sedentary	62.5 (20.5)	63.2 (20.3)	61.3 (21.2)	62.7 (20.0)
Daily regularity	19.0 (6.9)	19.0 (6.7)	18.6 (6.8)	19.5 (7.2)
Number of pets, N (%)				
0 pet	1203 (36.6)	515 (40.4)	336 (32.2)	352 (36.5)
1 pet	786 (23.9)	323 (25.4)	229 (21.9)	234 (24.2)
2 pets	604 (18.4)	216 (17.0)	224 (21.4)	164 (17.0)
3 or more pets	691 (20.9)	220 (17.4)	256 (24.5)	215 (22.3)
Fast food, N (%)				
No fast food	458 (13.9)	128 (10.0)	141 (13.5)	189 (19.6)
Once per month	1156 (35.2)	443 (34.8)	365 (34.9)	348 (36.1)
Once per week	1184 (36.1)	483 (37.9)	398 (38.1)	303 (31.4)
Several days per week	429 (13.1)	184 (14.4)	130 (12.4)	115 (11.9)
Nearly every day	57 (1.7)	36 (2.8)	11 (1.1)	10 (1.0)

Notes: ^a^ Variables measured in minutes.

**Table 2:** Correlations between various lifestyle factors and sleep health across the lifespan.

Lifestyle Factors	Young (n = 1274)	Middle (n = 1045)	Older (n = 965)
Fast food per week	−0.135 **	−0.126 **	−0.135 **
Number of pets	−0.063 *	−0.051	−0.010
Weekly MVPA minutes	0.022	0.075 *	0.090 **
Daily % sedentary	−0.021	−0.053	−0.102 **
Daily TV minutes	−0.132 **	−0.171 **	−0.129 **
Daily social media minutes	−0.131 **	−0.196 **	−0.163 **
Daily reading minutes	−0.066 *	−0.111 **	−0.023
Daily internet minutes	−0.152 **	−0.233 **	−0.093 **
Daily regularity	−0.320 **	−0.340 **	−0.283 **

Notes: MVPA = moderate to vigorous physical activity. * *p* < 0.05, ** *p* < 0.01.

**Table 3:** Age group comparisons of the associations between lifestyle factors and sleep health.

Lifestyle Factors	Young and Middle Comparisons		Young and Older Comparisons		Middle and Older Comparisons	
	Z	*p*	Z	*p*	Z	*p*
Fast food per week	−0.219	0.413	0.000	0.500	0.205	0.419
Number of pets	−0.288	0.387	−1.242	0.107	−0.918	0.179
Weekly MVPA minutes	−1.272	0.102	−1.597	0.055	−0.338	0.368
Daily % sedentary	7.670	0.222	**−1.904**	**0.028**	1.103	0.135
Daily TV minutes	0.955	0.170	−0.071	0.472	−0.961	0.168
Daily social media minutes	1.599	0.055	0.765	0.222	−0.763	−0.136
Daily reading minutes	1.085	0.139	−1.008	0.157	**−1.978**	**0.024**
Daily internet minutes	2.014	0.022	−1.402	0.080	**−3.223**	**0.001**
Daily regularity	0.537	0.296	−0.953	0.170	−1.412	0.079

Notes: MVPA = moderate to vigorous physical activity. Bolded values are statistically significant.

**Table 4:** Within age comparisons of the strength of association between lifestyle factors and sleep health.

	MVPA Minutes vs. Daily % Sedentary		Daily Social Media vs. Daily Internet Time	
	Z	*p*	Z	*p*
Younger adults	0.982	0.163	0.666	0.253
Middle-aged	**2.611**	**0.005**	1.043	0.148
Older adults	**3.691**	**0.001**	**−1.829**	**0.034**

Notes: MVPA = moderate to vigorous physical activity. Bolded values are statistically significant.

## Data Availability

The data presented in this study are available upon reasonable request from the corresponding author.
